# An AA9-LPMO containing a CBM1 domain in *Aspergillus nidulans* is active on cellulose and cleaves cello-oligosaccharides

**DOI:** 10.1186/s13568-018-0701-5

**Published:** 2018-10-17

**Authors:** Guru Jagadeeswaran, Lawrie Gainey, Andrew J. Mort

**Affiliations:** Department of Biochemistry and Molecular Biology, Oklahoma State University, Stillwater, OK 74078 USA

**Keywords:** Lytic polysaccharide monooxygenases, AN1602, AA9 LPMO, *Aspergillus nidulans*, Cellulose, Cellohexaose, Cello-oligosaccharides

## Abstract

Lytic polysaccharide monooxygenases (LPMOs) are copper dependent enzymes that carry out oxidative cleavage of cellulose and other polysaccharides. *Aspergillus nidulans,* an ascomycete fungus that contains multiple AA9 LPMOs in the genome, offers an excellent model system to study their activity during the oxidative degradation of biomass. AN1602, a dual domain AA9-LPMO in *A. nidulans* appended with a carbohydrate-binding module, CBM1, was expressed in *Pichia pastoris* for analyzing oxidative cleavage on cellulosic substrates. The mass spectral and HPAEC analyses showed that the enzyme cleaves phosphoric acid swollen cellulose (PASC) in the presence of a reducing agent, yielding a range of cello-oligosaccharides. In addition to the polymeric substrate cellulose, AN1602 is also active on soluble cellohexaose, a property that is restricted to only a few characterized LPMOs. Product analysis of AN1602 cleaved cellohexaose revealed that C4 was the sole site of oxidation. The sequence and predicted structure of the catalytic domain of AN1602 matched very closely to known C4 cellohexaose active enzymes.

**Electronic supplementary material:**

The online version of this article (10.1186/s13568-018-0701-5) contains supplementary material, which is available to authorized users.

## Introduction

Plant biomass degrading fungi secrete a diverse set of enzymes and significantly contribute to the global carbon cycle. Most of the polysaccharide degrading enzymes involved in biomass degradation break glycosidic bonds by hydrolysis. This then serves as the basis of how GH families are organized into the CAZY classification system. However, relatively recently lytic polysaccharide monooxygenases (LPMOs), a class of novel copper-dependent redox enzymes were discovered. These enzymes break glycosidic linkages by oxidation rather than hydrolysis. They make up a significant portion of plant cell wall degrading fungal secretomes (Floudas et al. 2012; Glass et al. 2013). It was first shown in 2010 that activity of cellulases on corn stover could be enhanced by the addition of LPMOs through an unknown mechanism (Harris et al. 2010). Later studies demonstrated fungal LPMOs act synergistically when combined with other glycoside hydrolases during the decomposition of crystalline cellulose to glucose monomers (Forsberg et al. 2011; Kim et al. 2014; Langston et al. 2011). To harness this potential in the conversion of lignocellulosic biomass, most modern commercial enzymatic cocktails, which are employed in biorefineries, contain LPMOs (Johansen 2016; Muller et al. 2015).

LPMOs, in spite of considerable variability in their primary sequences, share structural similarities. These include a surface exposed copper containing active site with a flat substrate-binding surface and a rigid β sheet core connected by flexible loops (Span and Marletta 2015). Molecular oxygen is generally considered to serve as a co-substrate during LPMO activity, although some recent reports indicate reactivity could be dependent on hydrogen peroxide (Bissaro et al. 2017). An electron donor, such as ascorbic acid, is required to activate molecular oxygen in the copper-containing active site (Beeson et al. 2012; Quinlan et al. 2011; Vaaje-Kolstad et al. 2010). In some cases, cellobiose dehydrogenase (CDH), a natural redox enzyme co-secreted with LPMOs by several fungi, has been shown to serve as a source of electrons (Langston et al. 2011; Phillips et al. 2011).

The position of carbon oxidations in the glycan chain varies, and LPMOs are classified accordingly. This classification scheme is broken into the three following regioselectivity classes: PMO1s act at C1; PMO2s at C4; and a third group (PMO3s) oxidize both at the C1 and C4 positions, which leads to the formation of either aldonic acid (C1) or 4-ketoaldose (C4) (Beeson et al. 2012; Hemsworth et al. 2013; Isaksen et al. 2014; Li et al. 2012; Vu et al. 2014).

In the carbohydrate active enzyme (CAZy) database, LPMOs are categorized under auxiliary activity (AA) families. Currently, they fall under AA9, AA10, AA11, AA13, AA14 and AA15 families based on sequence characteristics and substrate specificities (Couturier et al. 2018; Levasseur et al. 2013; Lombard et al. 2014; Sabbadin et al. 2018). AA9-LPMOs consist of fungal LPMOs that are mainly active on cellulose, and have been identified in the secretomes of both ascomycete and basidiomycete fungi during decomposition of lignocellulosic biomass (Berrin et al. 2017; de Gouvea et al. 2018; Ray et al. 2012; Saykhedkar et al. 2012; Znameroski and Glass 2013). In addition to being active on the insoluble cellulose, hemicelluloses (e.g. xyloglucan) have been shown to be amenable to LPMO activity (Agger et al. 2014; Bennati-Granier et al. 2015). The cleavage activity of LPMOs on xylan or xyloglucan, has been demonstrated when these hemicelluloses are coated onto cellulose (Frommhagen et al. 2015; Nekiunaite et al. 2016). Although the most common substrate for AA9-LPMOs remains to be insoluble cellulose, in limited instances, additional activity on small soluble cello-oligosaccharides has been reported (Bennati-Granier et al. 2015; Frandsen et al. 2016; Isaksen et al. 2014).

The genome of *A. nidulans*, an ascomycete fungus, contains nine genes encoding for LPMOs and offers an excellent model system to study their activity during oxidative degradation of biomass. Here we provide characterization of functional activity of AN1602, a two-domain LPMO in *A. nidulans,* that contains an N-terminal catalytic AA9 domain and a C-terminal family 1 carbohydrate-binding module (CBM1). Our studies show that AN1602 is capable of conducting oxidative cleavage on soluble cello-oligosaccharides in addition to cellulose. Our findings add to our understanding of substrate specificities and sequence/structure relationships of LPMOs.

## Materials and methods

### Production and purification of enzyme

In-frame cloning of the ORF corresponding to AN1602 from *A. nidulans* into the pPICZαC vector and its heterologous expression in *Pichia pastoris* X-33 (Invitrogen) were previously described (Bauer et al. 2006). The *Pichia* strain # 10071 (pPICZαC) is available from the Fungal Genome Stock Center http://www.fgsc.net/FGSCPichiaStrains.htm. Recombinant *Pichia* expressing AN1602 was cultivated in buffered glycerol-complex medium (BMGY) at 30 °C and cells were resuspended into buffered methanol-complex medium (BMMY) to induce AN1602 expression. Additional methanol (1%) was added to the media at 24 h intervals for two more days after induction and the protein was harvested in cultures grown for 72 h. The secreted AN1602 was concentrated 10–20 times using ultra-filtration, (10 kDa cutoff, Amicon, Danvers, MA, USA) or in sample concentration columns (MWCO 10000, GE Healthcare, Pittsburgh, PA, USA). One-milliliter fractions of the concentrated protein were further purified using size exclusion chromatography on Toyopearl HW 40S (1.0 × 17 cm) (Tosoh Biosciences, Tokyo, Japan) with a running buffer containing 50 mM ammonium acetate pH 5.2. The homogeneity of the protein was verified by SDS-PAGE.

Purified proteins were quantified by the Bradford method (Bradford 1976), and visualized in a SDS-PAGE gel (12.5%) after staining with Coomassie Blue R-250. The molecular mass of the proteins under denaturing condition was estimated by comparing to pre-stained protein standards (Biorad, Hercules, CA, USA). The tryptic digest of purified recombinant protein cut from an SDS-PAGE gel was used to verify protein identity by mass spectrometry (Additional file 1: Figure S1).

### Protein binding studies

Purified AN1602 protein (10 μg) was mixed with insoluble Avicel (2% w/v) in 20 mM sodium phosphate buffer, pH 6.0 in a final volume of 100 μL in 1.5 mL Eppendorf tubes. After incubating the tubes on ice for 3 h with gentle mixing, the unbound proteins in the supernatants was carefully removed by centrifuging at 10,000×*g* for 5 min. The pellet was washed twice by resuspending in sodium phosphate buffer and the second wash was used for verifying the wash fraction. To release the bound protein, the pellet was resuspended in SDS-loading buffer and was denatured by boiling for 5 min (with a volume equivalent to the unbound fraction). All sample fractions were verified on the SDS-PAGE gel.

### Glycosylation

Glycosylation sites were predicted using NetNGlyc 4.0 (for *N*-glycosylation) (http://www.cbs.dtu.dk/services/NetNGlyc/) and NetOGlyc 3.1 (for *O*-glycosylation) (http://www.cbs.dtu.dk/services/NetOGlyc/). Recombinant AN1602 was deglycosylated with PNGase F as per manufacturer’s procedure (New England Biolabs, Évry, France) under denaturing conditions followed by mobility shift analysis using SDS-PAGE gel.

### Sequence alignment

A sequence alignment of AN1602 with biochemically characterized LPMOs from other species was performed using the Clustal Omega multiple sequence alignment program (http://www.ebi.ac.uk/Tools/msa/clustalo/). A structural homology model for AN1602 was generated using the I-TASSER Suite server (http://zhanglab.ccmb.med.umich.edu/I-TASSER/download/) with the crystal structure of LsAA9A (PDB-id: 5ACFA) as template with a sequence identity of 0.51 in the threading aligned region with the query sequence.

### Enzyme activity

Cellulose cleavage assays were carried out in 50 mM sodium acetate buffer pH 5.0 containing 0.1% (w/v) PASC, 1.0 μM of AN1602 and 1 mM ascorbate as a reducing agent. Phosphoric acid-swollen cellulose (PASC) was prepared from Avicel PH-101 (Sigma-Aldrich, St. Louis, MO, USA) as described in Wood (1988). For assessing cello-oligosaccharide degradation, standard reaction mixtures containing 0.5 mg/mL cellohexaose were incubated under similar conditions to that of PASC. The reaction mixtures were shaken at 800 rpm in an Eppendorf thermomixer (Eppendorf, Montesson, France) at 45 °C for 24 h, and further enzymatic activity was stopped by heating to 100 °C for 5 min. The soluble fraction from the mixture was separated by centrifuging at 13,000 RPM for 5 min and stored at 4 °C until used for analysis.

### MALDI-TOF MS

After enzymatic incubation, degradation products from each sample were assayed by MALDI-TOF MS (matrix-assisted laser desorption/ionization-time of flight mass spectrometry). Prior to analysis, samples were desalted via graphitized carbon packed into a pipette tip using 50% V/V acetonitrile/water as eluent (Redmond and Packer 1999). The desalted samples were spotted in replicates onto target plates (Perseptive Biosystems, Framingham, MA, USA), mixed with a matrix (9 mg/mL solution of 2,5-dihydroxybenzoic acid (DHB) in 30% acetonitrile) in 1:1 ratio, and air dried. MALDI-TOF MS analysis was performed on a Voyager-DE Pro (Applied Biosystems, Foster City, CA, USA). For mass calibration of the instrument, a standard mixture of maltodextrins in a mass range of 300–2000 m/z was used. The instrument was operated in the positive ion reflector mode. The spectra for each test sample were collected by averaging multiple laser shots fired at the lowest energy that gave the best signal to noise ratio. The data were processed in Voyager Data Explorer processing software (ABI Voyager System, Foster City, CA, USA).

### Mass spectrometry

Electrospray ionization mass spectrometry (ESI–MS) was performed with a hybrid LTQ-Orbitrap mass spectrometer (Thermo Fisher Scientific, San Jose, CA USA) coupled to a PV 550 (New Objective) nano-electrospray ion source and an Eksigent NanoLC-2-D chromatography system. Reaction products were diluted in HPLC Grade water (Honeywell Burdick & Jackson) containing 0.1% formic acid (Honeywell) and aliquots of 10 µl of sample were injected into the chromatographic system. Chromatographic separation was accomplished with a 5 cm length × 150 µm ID pre-column followed by analytical separation on an 18 cm length × 75 µm ID fused silica column packed in-house with Magic AQ (C18, 3 µm). Both columns terminated with an integral fused silica emitter pulled in house. Gradient elution was carried out with water/0.1% formic acid (solvent A) and 80% acetonitrile/20% water containing 0.1% formic acid (solvent B). Samples were eluted using a 50–87.6% Solvent B gradient performed over 30 min, followed by 87.6–100% Solvent B gradient performed over 30 min, and held at 100% Solvent B for the reminder of the 76 min run. All flow rates were 250 nl/min. Precursors were selected during the full-range FT-MS scan (nominal resolution of 60,000 FWHM, 360–2000 m/z) and data were collected in positive (PI) mode. MS/MS settings used a trigger threshold of 8000 counts and rejection of parent ions that were previously selected for MS/MS. Data dependent acquisition was carried out using a dynamic exclusion for 150% of the observed chromatographic peak width. The most intense ions were selected, fragmented, and analyzed in the linear ion trap using four subsequent data dependent MS/MS scans at normalized energy levels of 40, 50, 60, or 70. Fragmentation occurred using collisional induced dissociation (CID) energies with ultra-high purity (99.9%) helium as the collision gas. Blank injections were performed between samples to assay for contamination and to minimize sample-to-sample chromatographic contamination. Data was visualized using Xcalibur 3.0.63 (Thermo Scientific).

### PACE analysis

For PACE, reaction products and oligosaccharide standards (Megazyme) were reductively aminated with 8-aminopyrene-1,3,6-trisulfonate (APTS) and separated by acrylamide gel electrophoresis in 20% resolving and 8% stacking gels following standard protocols (Goubet et al. 2011). For labelling, APTS was used, instead of 8-aminonaphthalene-1,3,6-trisulfonic acid (ANTS), because the typhoon fluorescence imager was not equipped with the UV excitation needed for ANTS. PACE was performed using a 192 mM glycine, 25 mM tris, pH 8.5 running buffer. All electrophoresis was carried out in cold room at 4  °C. The gel was visualized at an excitation wavelength set to 488 nm in a Typhoon fluorescence imager equipped with a Cy2 520 nm fluorescence detection filter. Experiments were repeated and a representative gel sample is presented.

### HPAEC analysis

Reaction products were analyzed by high-performance anion exchange chromatography (HPAEC) with pulsed amperometric detection (PAD) following (Isaksen et al. 2014) with minor modifications. The HPAEC system (BioLC, Dionex, Sunnyvale, CA, USA) was equipped with a electrochemical gold electrode and CarboPac PA1 analytical column (250 mm × 2 mm i.d., Dionex). Ten microlitre reaction samples were injected, and eluted using a gradient of 0.1 M NaOH (eluent A) and 1 M NaOAc in 0.1 M NaOH (eluent B). Solute elution was performed at 0.3 mL/min with initial conditions set to 0.1 M NaOH (100%). A stepwise linear gradient was applied as follows: a 10 min linear gradient from 100% eluent A (starting condition) to 10% eluent B, a 15 min linear gradient to 30% eluent B, and a 5 min gradient to 100% eluent B. The column was reconditioned between each run by running initial conditions for 10 min. Non-oxidized cello-oligosaccharides were used as standards.

## Results

### Heterologous expression of the recombinant AN1602

**Fig. 1:**
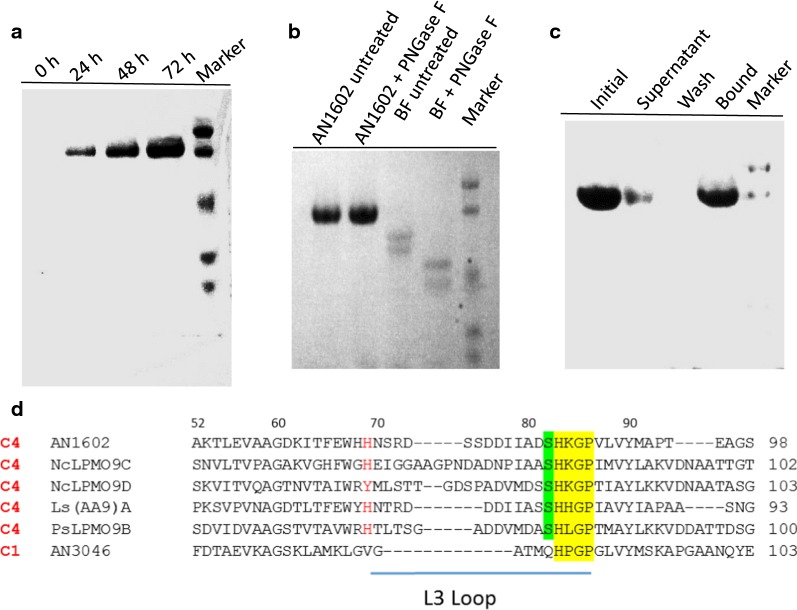
Production of AN1602 in *P. pastoris* and analysis. **a** Time course of AN1602 secretion over a period of 3 days from *P. pastoris* cultures after induction with 1.0% methanol. Aliquots of *P. pastoris* culture media taken at 24 h intervals were assessed in SDS-PAGE gel under denaturing conditions. **b** SDS-PAGE analysis of purified AN1602. AN1602 protein was treated with PNGase F and loaded onto a denaturing PAGE gel as follows: purified AN1602 (lane1), AN1602 after PNGase F treatment (lane 2), bovine feutin (BF) (lane 3), BF after PNGase F treatment (lane 4), Marker—low molecular protein standard. Control reactions with BF in lane 3 and lane 4 shows the PNGase F enzyme is effective in deglycosylating BF. **c** Binding of AN1602 to insoluble cellulose. AN1602 protein was mixed with Avicel and incubated on ice for 3 h in sodium phosphate buffer and the PAGE gel was loaded as fractions containing initial starting material (lane 1), supernatant (lane 2), wash (lane 3) and protein bound to pellet (lane 4). Marker—low molecular protein standard. **d** Sequence features of AN1602. A partial multiple sequence alignment of selected LPMOs. The conserved [Hx_n_GP] motif containing the second His ligand of the copper active site is highlighted in yellow, and the conserved serine in green. The predicted L3 loop region is marked by a horizontal bar and the first residue of the loop is shown in red. Regioselectivity is (C1/C4) is indicated on the left based on published literature (Frandsen et al. 2016; Isaksen et al. 2014; Jagadeeswaran et al. 2016)

Our previous transcriptome analysis of *A. nidulans* identified nine putative LPMO sequences. Of these, AN1602, an AA9-LPMO abundantly induced by cellulose, contains a carbohydrate binding module 1 (CBM1) in addition to the catalytic domain (Jagadeeswaran et al. 2016). The ORF of AN1602 encodes a putative protein of 357 amino acids with a preponderance of alanine, serine and threonine residues and includes a secretion signal peptide of 18 amino acids (MKFSSVLALAASAKLVAS). We expressed the protein from a AN1602 cDNA previously cloned into the pPICZαC expression vector (Bauer et al. 2006) for subsequent expression in yeast *P. pastoris*. The culture supernatant, harvested after a period of 3 days of methanol induction, showed specific induction of the AN1602 protein (Fig. 1a). Low molecular weight components from the medium were removed by gel filtration chromatography (Additional file 1: Figure S1a). The identity of the protein was verified by LC–MS/MS that showed extensive coverage by tryptic peptides from the catalytic domain and CBM (Additional file 1: Figure S1b). In the linker region, tryptic peptides are predicted to be very long and likely to be heavily glycosylated, thus would not be recognized by the proteomics software. We also found tryptic peptides matching the *A. nidulans* signal peptide. It is likely that the secreted AN1602 contained a mixture of correctly processed mature protein with the N-terminal His residue needed for activity, and a fraction retaining the native signal peptide.

When resolved on a PAGE gel, the protein representing AN1602 appeared to be homogeneous as an ~ 70 kDa band, a molecular mass higher than the predicted mass of 36.7 kDa based on the protein sequence (Fig. 1b). The observed difference in the protein mass likely arises from glycosylation that has been seen in several LPMOs. The *N*-glycosylation prediction server, NetNGlyc, predicted no *N*-glycosylation sites to be present in the protein. This was supported by the treatment of AN1602 with PNGase F, an enzyme that acts on *N*-glycosylated residues, and showed no observable change in migration pattern of the band under denaturing conditions (Fig. 1b). Analyzing the protein for possible *O*‐glycosylation, the NetOGlyc server predicted many sites on the serine and threonine residues in the linker region (residues 235–318), located between the catalytic and the CBM1 module of the enzyme (Additional file 1: Figure S1c). Since PNGase F does not deglycosylate O-linked glycans, we suspect that O-linked glycosylations are contributing to the observed higher mass of the protein.

In order to test if the CBM1 module has cellulose-binding characteristics, we analyzed the binding properties of the AN1602 by incubating purified AN1602 with Avicel cellulose (Fig. 1c). The unbound protein in the supernatant was removed after 3 h. Then, the pellet bound protein was washed twice and finally eluted by denaturing the protein. Samples from the supernatant, the wash, and the bound fractions were checked qualitatively on an SDS-PAGE gel. Some protein was found to remain unbound in the supernatant. However, most of the AN1602 was recovered from the Avicel-bound fraction obtained after denaturing the protein. The wash fraction did not contain any protein. These data suggest binding of AN1602 to cellulose is not due to non-specific binding (Fig. 1c), although it cannot be ascertained that the binding was specifically mediated by the cellulose-binding domain belonging to family 1 of CBM.

Sequence analysis of the N-terminal catalytic domain showed AN1602 has a terminal histidine ligand for copper, and a second active site histidine ligand contained in an [Hx_n_GP] motif, typical of most LPMOs (Fig. 1d, Additional file 1: Figure S2a). Of the three regioselectivity classes, the presence of a surface serine residue immediately before the second copper active site histidine ligand in the [HxnGP] motif, as seen in AN1602, is characteristic of PMO2s (Fig. 1d; (Beeson et al. 2015; Vu et al. 2014). A multiple sequence alignment of well-characterized C4-specfic PMO2s (Frandsen et al. 2016; Isaksen et al. 2014; Patel et al. 2016), along with a C1 oxidizing PMO1 [AN3046; (Jagadeeswaran et al. 2016)], showed AN1602 has additional features representative of PMO2s. This includes the presence of a lysine residue immediately following the second histidine ligand, and an insertion referred to as the L3 loop located between beta strands on the substrate-binding surface. In all solved AA9-LPMO structures, loop L3 is the most variable and is relatively longer (by about 9–14 residues) in PMO2s than in PMO1s (Frandsen and Lo Leggio 2016; Lo Leggio et al. 2012; Meier et al. 2018). Accordingly, the region corresponding to the L3 loop in AN1602, beginning at a His residue conserved in PMO2s and ending a few residues before the second copper His ligand, is longer and has an aromatic residue (histidine) as the first residue of the L3 loop (Fig. 1d), features indicative of C4 oxidizing LPMOs (Borisova et al. 2015; Liu et al. 2018).

### Enzyme activity on cellulose and cello-oligosaccharides

**Fig. 2:**
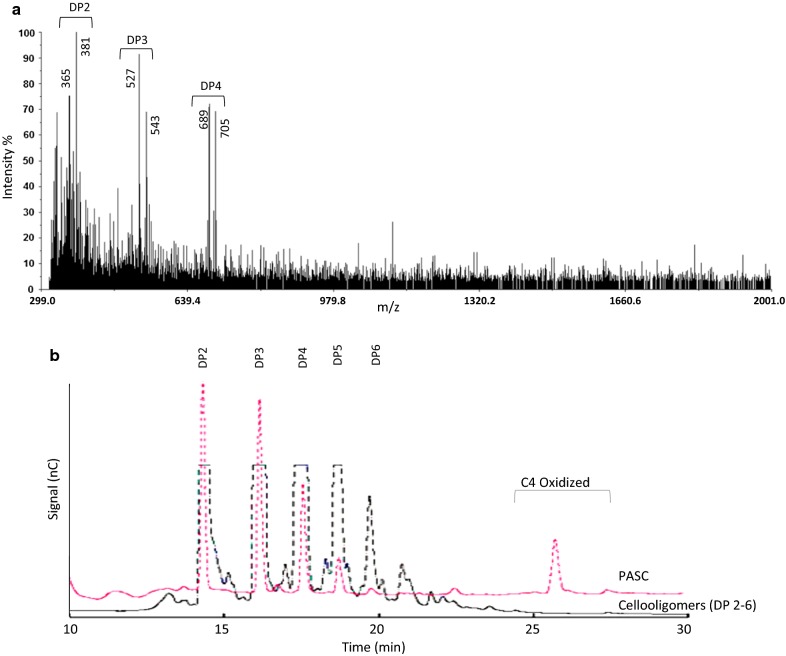
Activity of AN1602 on amorphous cellulose (PASC). **a** MALDI-ToF spectra of products generated from the incubation of PASC with AN1602. Avicel (1%, w/v) was incubated with AN1602 (1 µM) in 50 mM sodium acetate (pH 5.5) in the presence of 1 mM of ascorbic acid at 45 °C for 24 h. **b** HPAEC chromatogram showing the generation of native and oxidized oligosaccharides after incubation of PASC with AN1602. A mixture of non-oxidized cello-oligosaccharide (DP2-6) standard as control run for peak annotation is also shown. Analysis was carried out after treating PASC under similar conditions as described in **a**. *nC* nanocoulomb

In order to test if AN1602 expressed in *Pichia* is functional, initial screening of the enzyme activity towards cellulose was carried out with matrix assisted laser desorption ionization time-of-flight mass spectrometry (MALDI-TOF MS) analysis. For this, polymeric substrate phosphoric acid swollen cellulose (PASC) was incubated with purified AN1602 added to a final concentration of 1 µM and the soluble reaction products released after 24-h incubation were analyzed by mass spectrometry (Fig. 2a). A product profile primarily corresponding to DP2 through DP4 was detected. Ions of *m/z* 365 (DP2), 527 (DP3) and 689 (DP4) corresponding to sodium adducts of native cello-oligosaccharides [M + Na]^+^, and m/z values 381 (DP2), 543 (DP3), and 705 (DP4) corresponding to their respective oxidized cello-oligosaccharides [M + 16 + Na]^+^, were seen (Fig. 2a).

We further subjected the samples obtained from the digestion of PASC with AN1602 to HPAEC-PAD (high-performance anion-exchange chromatography with pulsed amperometric detection) analysis. As observed with MALDI-TOF MS, profiles observed in HPAEC-PAD showed release of soluble cello-oligosaccharides corresponding to DP2, DP3 and DP4, and also later eluting peaks (between 26 and 28 min) potentially corresponding to C4-oxidized species (Fig. 2b). Annotation of oxidized species in HPAEC-generated chromatograms was based on published elution patterns of C1/C4-oxidized cello-oligosaccharides (Bennati-Granier et al. 2015; Isaksen et al. 2014; Westereng et al. 2017). Chromatogram patterns did not detect any peaks corresponding to aldono-lactone products (C1 oxidation). Enzyme reactions without ascorbic acid did not show formation of oxidized or native cello-oligomers (data not shown).

**Fig. 3:**
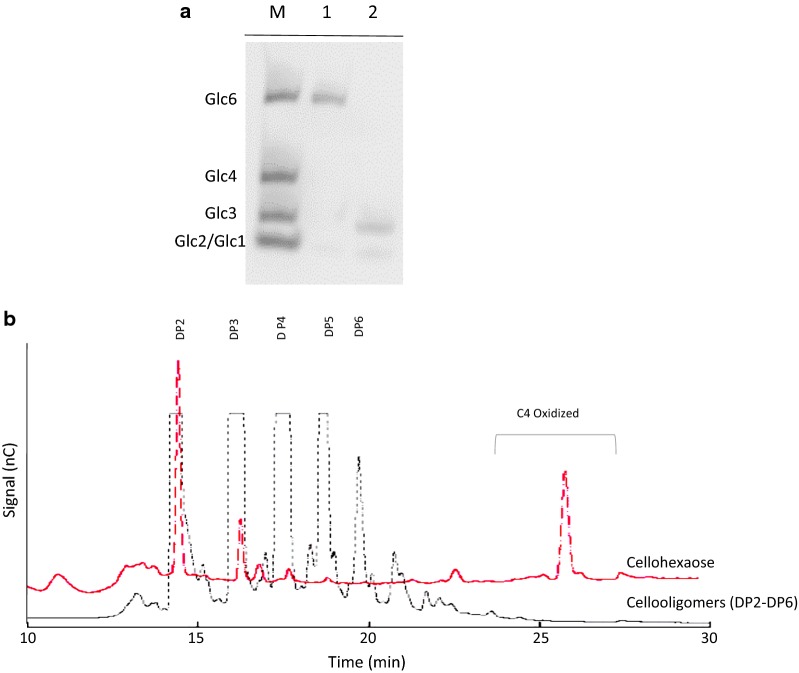
Activity of AN1602 on cellohexaose. **a** PACE gel showing digestion products from cellohexaose upon AN1602 activity in the presence of 1 mM ascorbic acid. Lane 1—cellohexaose, lane 2—cellohexaose treated with AN1602. The migration standards (M) are cello-oligosaccharides corresponding to Glc2 (cellobiose), Glc3 (cellotriose), Glc4 (cellotetraose) and Glc6 (cellohexaose). **b** HPAEC chromatogram showing the generation of native and oxidized oligosaccharides after incubating cellohexaose (0.5 mg/mL) with AN1602. A mixture of nonoxidized cello-oligosaccharide (DP2-6) standards in the control run is also shown. Analysis was carried out after treating cellohexaose with AN1602 under similar conditions as in Fig. 2a. *nC* nanocoulomb

In order to assess the cleavage potential of AN1602 on soluble cello-oligosaccharides, we incubated the enzyme with cellohexaose (Glc6) in the presence of the reducing agent, ascorbic acid, and examined the product profile using carbohydrate electrophoresis (PACE) (Fig. 3a). Analysis of digested products separated by acrylamide gel electrophoresis showed AN1602 is active on soluble cellohexaose yielding cellotriose (Glc2) and cellobiose (Glc3) (Fig. 3a). To further test the digested products from the activity of AN1602, we subjected the reaction products to HPAEC analysis. The elution patterns of digested products from cellohexaose showed peaks corresponding to native cellobiose and cellotriose and later eluting peaks potentially corresponding to their C4 oxidized counterparts (Fig. 3b). The absence of degradation products after incubation without the reducing agent (results not shown), further confirmed AN1602 is capable of acting on soluble cello-oligosaccharides.

### MS–MS analysis

**Fig. 4:**
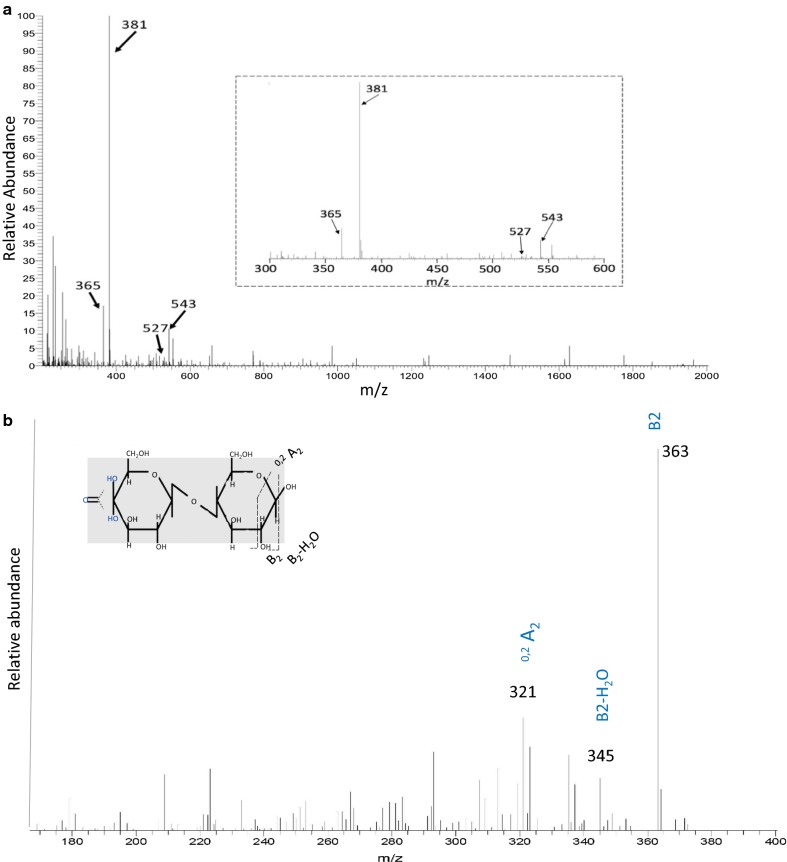
Mass spectrometry analysis of degradation products generated by AN1602 from cellohexaose. **a** The panel shows full spectrum of sample with peaks corresponding to native and oxidized cello-oligosaccharides after 24 h of cellohexaose degradation by AN1602.The inset shows the close-up of the spectra in the m/z 300–600. **b** MS/MS spectra of oxidized product with m/z 381 that was further fragmented. Masses are labeled based on expected fragmentation from the C4 oxidized product Glc4gemGlc. The inset shows the likely position of fragmentation products from Glc4gemGlc

To further verify the mode of action of AN1602 on cello-oligosaccharides, we analyzed the hydrolysate obtained from incubation of cellohexaose by capillary reverse phase HPLC mass spectrometry (Fig. 4). The spectrum showed two groups of peaks corresponding to cello-oligosaccharides of DP2 and DP3. The major peaks for DP2 and DP3 correspond to oxidized Glc2 at m/z 381 and oxidized Glc3 at m/z 543. A considerable amount of non-oxidized Glc2 (m/z 365) and a minor amount of non-oxidized Glc3 (m/z 527) were also observed (Fig. 4a).

For determining the oxidative regioselectivity of AN1602 on the substrate, and to verify the position of oxidation either at C1 or C4-carbon position, MS/MS mass spectrometry analysis was performed on oxidized DP2-(ions at m/z 381) and DP3-(ions at m/z 543) product species (Fig. 4b and Additional file 1: Figure S3). The MS^2^ fragmentation spectrum of m/z 381 contained two major fragments, B_2_ and B_2_-H_2_O. Fragment B2 (m/z 363), arising from the loss of one water molecule (m/z 381 − 18), and B_2_-H_2_O (m/z 345), arising from the loss of two water molecules (m/z 381 − 36) from gem-diol of Glc2, are typical of C4-oxidation of cellobiose. Glc2ox (keto) generated from C4 specific regioselective activity AN1602, would readily hydrate in a nonenzymatic chemical step to form a geminal diol in aqueous medium (Kopper and Freimund 2003). Additional fragments prevalent in the spectra arising from cross ring cleavages at ^0, 2^A2 (m/z 321), were further indicative of gem-diol oxidation (Fig. 4b). Similar to the MS^2^ fragmentation patterns of m/z 381, fragmentation of DP3-oxidized product showed the prevalence of two fragments corresponding to B_3_ (m/z 525) and B_3_-H_2_O (m/z 507) cleavages; ions distinctive of C4 oxidation (Additional file 1: Figure S3). Analyses of fragmentation spectra in MS^2^ were based on the expected cleavages arising from oxidized DP2 and DP3 as given by Isaksen et al. (2014).

### Structural model of AN1602 and sequence analysis

**Fig. 5:**
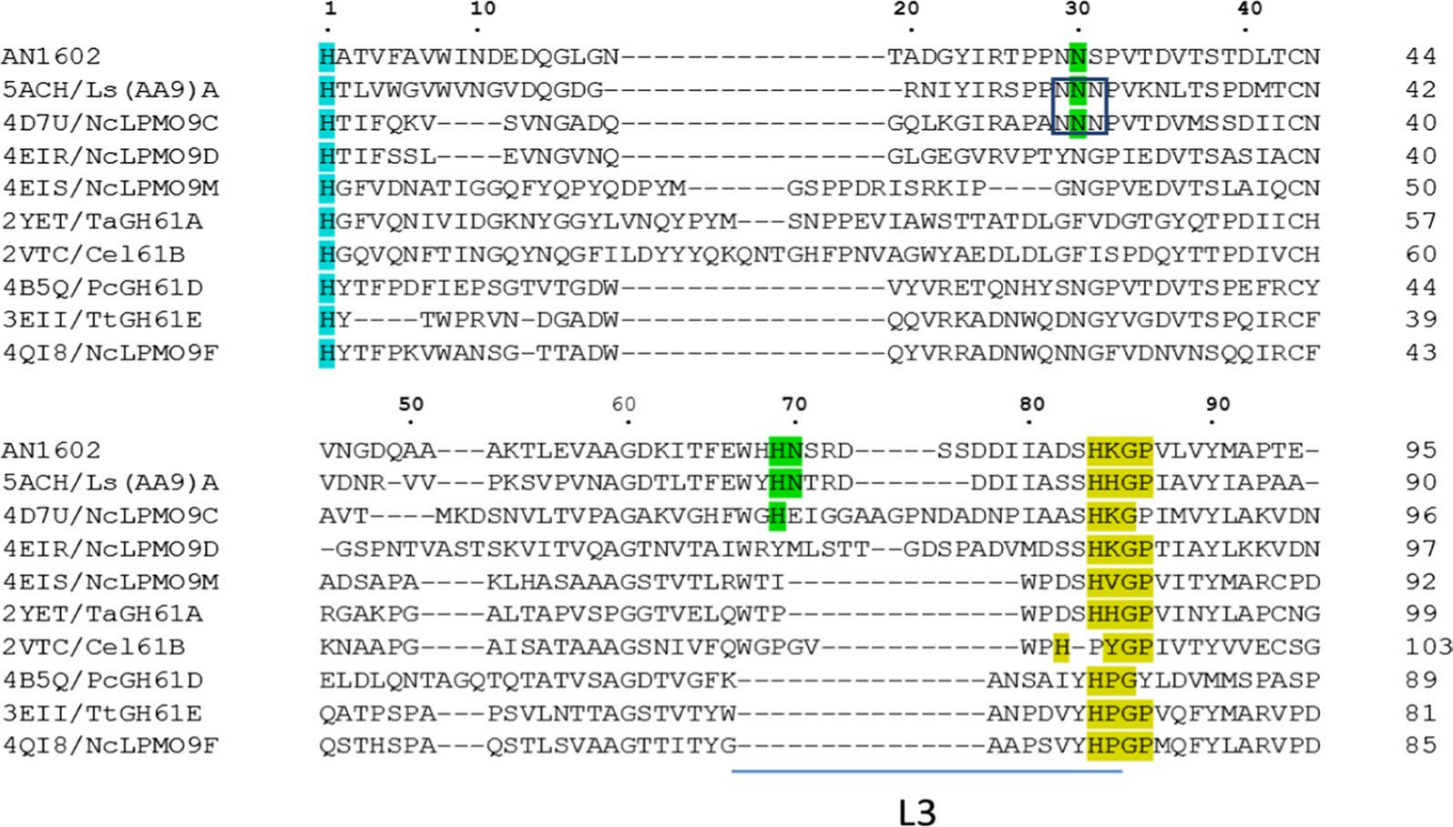
Sequence alignment of AN1602 and AA9-LPMOs with known structures. The proteins included (PDB ID in parenthesis) are: *Ls*(AA9)A (5ACH), *Nc*LPMO9C (4D7U), *Nc*LPMO9D (4EIR), *Nc*LPMO9M (4EIS), *Ta*GH61A (2YET), Cel61B (2VTC), *Pc*GH61D (4B5Q) *Tt*GH61E (3EII) and *Nc*LPMO9F (4QI8). Conserved residues Asn30, His69 and His70 in AN1602 and their equivalent residues in *Ls*(AA9)A and *Nc*LPMO9C that potentially determine the activity on cello-oligomers are highlighted in green. Conserved His1 is shown in turquoise. Cluster of three consecutive aspargine residues in NcLPMO9C and Ls(AA9)A is boxed. Residues corresponding to Insertion loop L3 is shown as a horizontal bar below the sequence

In order to find any unique features potentially associated with the ability of AN1602 to act on cello-oligosaccharides, we compared the protein sequence of AN1602 with LPMOs whose 3D structures had been determined. Sequence analyses showed AN1602 is most closely related to *Ls*(AA9)A, followed by *Nc*LPMO9C with 51.1% and 43.8% amino acid identities, respectively (Additional file 1: Table S1). Some features previously thought to be of importance for activity on soluble cello-oligomers are conserved between AN1602 and these two LPMOs (Fig. 5). In AN1602 residues Asn30, His69 and Asn70, which are implicated in anchoring oligosaccharide substrates via hydrogen bonding (Frandsen et al. 2016), are conserved between the three LPMOs except the Asn70 equivalent in *Nc*LPMO9C is a Glu. The Asn30 equivalent residue is flanked by an asparagine on either side in both *Nc*LPMO9C and *Ls*(AA9)A, where the cluster of three consecutive Asn residues exposed at the surface was suggested to play a role in interacting with oligosaccharides (Fig. 5, (Isaksen et al. 2014). In AN1602 the Asn30 is flanked by asparagine and serine. AN1602 contains an L3 loop that is generally absent in LPMOs that do not degrade cello-oligosaccharides (Fig. 5, Additional file 1: Figure S4).

## Discussion

Approximately 20% of the fungal AA9 LPMOs contain a C-terminal CBM1 (Harris et al. 2010), and to date, only a few fungal AA9 LPMOs containing a CBM1 module have been characterized. Of the nine LPMO sequences in *A. nidulans*, AN1602, an AA9-LPMO abundantly induced by cellulose, contains a CBM1 in addition to the catalytic domain (Jagadeeswaran et al. 2016). LPMOs harboring a CBM1 module were shown to exhibit higher cellulose degradation ability than those without (Bennati-Granier et al. 2015; Crouch et al. 2016). Therefore AN1602 was selected for further characterization. Our data show AN1602 features a bimodular structure. It contains a recognizable, and experimentally functional, cellulose-binding domain (319–355 aa; Additional file 1: Figure S2) belonging to family 1 of the CBMs in the CAZy database, and has a functional catalytic AA9 (19–294 aa) domain capable of acting on cello-oligosaccharides in addition to cellulose.

Three regioselective groups of AA9 LPMOs (PMO1s oxidizing C1, PMO2s oxidizing C4, and PMO3s oxidizing both C1 and C4) have been proposed based on sequence alignments (Beeson et al. 2015; Vu et al. 2014). While regioselectivity may not be fully predictable by sequence data alone, several sequence/structural features of AN1602 correlate with aspects suggested to be important for C4 oxidizing activity of the enzyme (Fig. 1d). Analysis of enzyme activity towards polymeric cellulose (PASC) with HPAEC-PAD showed AN1602 is a functional LPMO and belongs to type 2 PMO (oxidation at the C4 position), in agreement with the prediction made based on sequence analyses. The fact that C4-oxidized gluco-oligosaccharides are highly unstable under the alkaline conditions employed in the HPAEC analysis chromatographic conditions likely contributed to the low recovery of oxidized species (Fig. 2, Frommhagen et al. 2017). It is also possible that some of the peaks corresponding to native cello-oligosaccharides might actually have arisen from C4-oxidized species that have lost the unstable 4-keto-glucose at the non-reducing end as shown by (Westereng et al. 2017).

While the MALDI and HPAEC analyses clearly demonstrated the functionality of AN1602 on PASC (Fig. 2), the degradation profile was distinctly different from most LPMOs characterized so far. LPMOs, when incubated with cellulose, generally yield longer oligosaccharides with DP up to 6 or 7. In contrast to this, predominantly shorter oligosaccharides, including native and oxidized cellobiose (DP2), were recovered in the degradation profiles of AN1602 indicating that the enzyme could possibly act on soluble cello-oligosaccharides, an activity hitherto shown only for a few LPMOs. Experimental evidence from PACE and mass spectrometry experiments confirmed AN1602 is indeed capable of acting on soluble cello-oligosaccharides (Figs. 3 and 4). Activity of AN1602 on cellohexose showed two groups of peaks corresponding to cello-oligosaccharides of DP2 and DP3 in both HPAEC and MS/MS mass spectrometry analysis. The MS^2^ fragmentation spectrum of m/z 381 and m/z543 provided further evidence that AN1602 is a PMO2 type LPMO. Because only a gem-diol formed at the non-reducing end leads to a double loss of water (B_2_-H_2_O and B_3_-H_2_O), observed in the fragmentation spectra of both m/z 381 and m/z543 (Fig. 4 and Additional file 1: Figure S3, Isaksen et al. 2014), C4 is the sole site of oxidative cleavage of cellohexose by AN1602.

Activity on soluble cello-oligosaccharides has only been reported for a limited number of AA9-LPMOs, and is important for facilitating investigations of enzyme–substrate interactions (Bennati-Granier et al. 2015; Courtade et al. 2016; Frandsen et al. 2016; Isaksen et al. 2014). Our analyses comparing AN1602 with AA9-LPMOs whose crystal structures have been determined showed that the enzyme is most closely related to *Ls*(AA9)A, followed by *Nc*LPMO9C, both with demonstrated ability to degrade cello-oligosaccharides (Additional file 1: Table S1, Frandsen et al. 2016; Isaksen et al. 2014). In addition, certain features previously thought to be of importance for activity on soluble cello-oligomers are conserved between AN1602 and these two LPMOs (Fig. 5). Of particular interest are the three residues Asn30, His69 and Asn70, which are implicated in anchoring oligosaccharide substrates via hydrogen bonding (Frandsen et al. 2016). In fact, only AN1602 showed conservation of all three (Asn30, His 69 and His70), whereas in NcLPMO9C two (Asn30, His 69) residues were conserved. None of the LPMOs where these residues are absent had any known cello-oligosaccharide activity. The cluster of three consecutive Asn residues exposed at the surface in NcLPMO9C (corresponding to Asn29, Asn30 and Ser31 in AN1602) and proposed to play a role in interacting with oligosaccharides (Isaksen et al. 2014) was not conserved in AN1602. A similar case was observed for *Pa*LPMO9H, another LPMO capable of acting upon cello-oligomers wherein the first and third asparagine residues are substituted by Ser and Phe (Bennati-Granier et al. 2015). In both *Pa*LPMO9H and AN1602, however, the central Asn30 is conserved suggesting that the central Asn30 is sufficient for exerting activity on soluble cello-oligomers as observed by Bennati-Granier et al. (2015). Besides the noted residues, loop L3, an insert just before the second conserved histidine copper-binding site, has been suggested to be involved in substrate-binding and determining activity on soluble substrates (Borisova et al. 2015). Only C4 oxidizing LPMOs contain a prominent L3 insert. No strictly C1 oxidizing LPMOs (which generally lack the insert) have been shown to degrade oligosaccharides. As such, our work provides further evidence that LPMOs are constrained in their ability to degrade soluble oligosaccharides by sequence and structural aspects of the protein. Lack of soluble substrate active LPMOs is one of the primary constraints in conducting kinetic studies on LPMO activity. Addition of AN1602 to the limited repertoire of cello-oligosaccharide active LPMOs should contribute to elucidation of kinetics of substrate oxidation by these enzymes.

## Additional file

**Additional file 1: Table S1.** Amino-acid identities (%) of AN1602 with other LPMOs. Alignments were performed using ClustalW using the catalytic domains of LPMO9s. **Figure S1.** Identification of secreted protein from *Pichia* culture media. **Figure S1a.** Protein elution profiles of AN1602 expressed in *P. pastoris* during gel filtration. **Figure S1b.** Sequence of identified protein using orbitrap mass spectrometry. Matched peptides observed in the spectrum are shown in yellow. Oxidized and alkylated residues are highlighted in green. **Figure S1c.** Predicted O-glycosylation sites (highlighted in green) in AN1602 from NetOGlyc server.**Figure S2a.** Protein sequence of AN1602. **Figure S2b.** Modular organization of AN1602 showing signal peptide (SP), catalytic (AA9), linker and CBM1 domains; numbers represent amino acid residues.**Figure S3.** MS/MS spectra of oxidized product with m/z 543 that was further fragmented. Masses are labeled based on expected fragmentation from the C4 oxidized product oxidized Glc3. **Figure S4.** Structure-guided homology model of AN1602 obtained from a structural overlay on the crystal structure of Ls(AA9)A (purple template, PDB code: 5ACF).
