# Supplementary material for: An AA9-LPMO containing a CBM1 domain in Aspergillus nidulans is active on cellulose and cleaves cello-oligosaccharides
Source: AMB Express. 2018 Oct 17;8:171. doi: 10.1186/s13568-018-0701-5 (PMC6192940; doi:10.1186/s13568-018-0701-5)
Supplement: Supplementary file 1 — Additional file 1: Table S1. Amino-acid identities (%) of AN1602 with other LPMOs. Alignments were performed using ClustalW using the catalytic domains of LPMO9s. Figure S1. Identification of secreted protein from Pichia culture media. Figure S1a. Protein elution profiles of AN1602 expressed in P. pastoris during gel filtration. Figure S1b. Sequence of identified protein using orbitrap mass spectrometry. Matched peptides observed in the spectrum are shown in yellow. Oxidized and alkylated residues are highlighted in green. Figure S1c. Predicted O-glycosylation sites (highlighted in green) in AN1602 from NetOGlyc server.Figure S2a. Protein sequence of AN1602. Figure S2b. Modular organization of AN1602 showing signal peptide (SP), catalytic (AA9), linker and CBM1 domains; numbers represent amino acid residues.Figure S3. MS/MS spectra of oxidized product with m/z 543 that was further fragmented. Masses are labeled based on expected fragmentation from the C4 oxidized product oxidized Glc3. Figure S4. Structure-guided homology model of AN1602 obtained from a structural overlay on the crystal structure of Ls(AA9)A (purple template, PDB code: 5ACF). [file 13568_2018_701_MOESM1_ESM.pptx]

## Slide 1
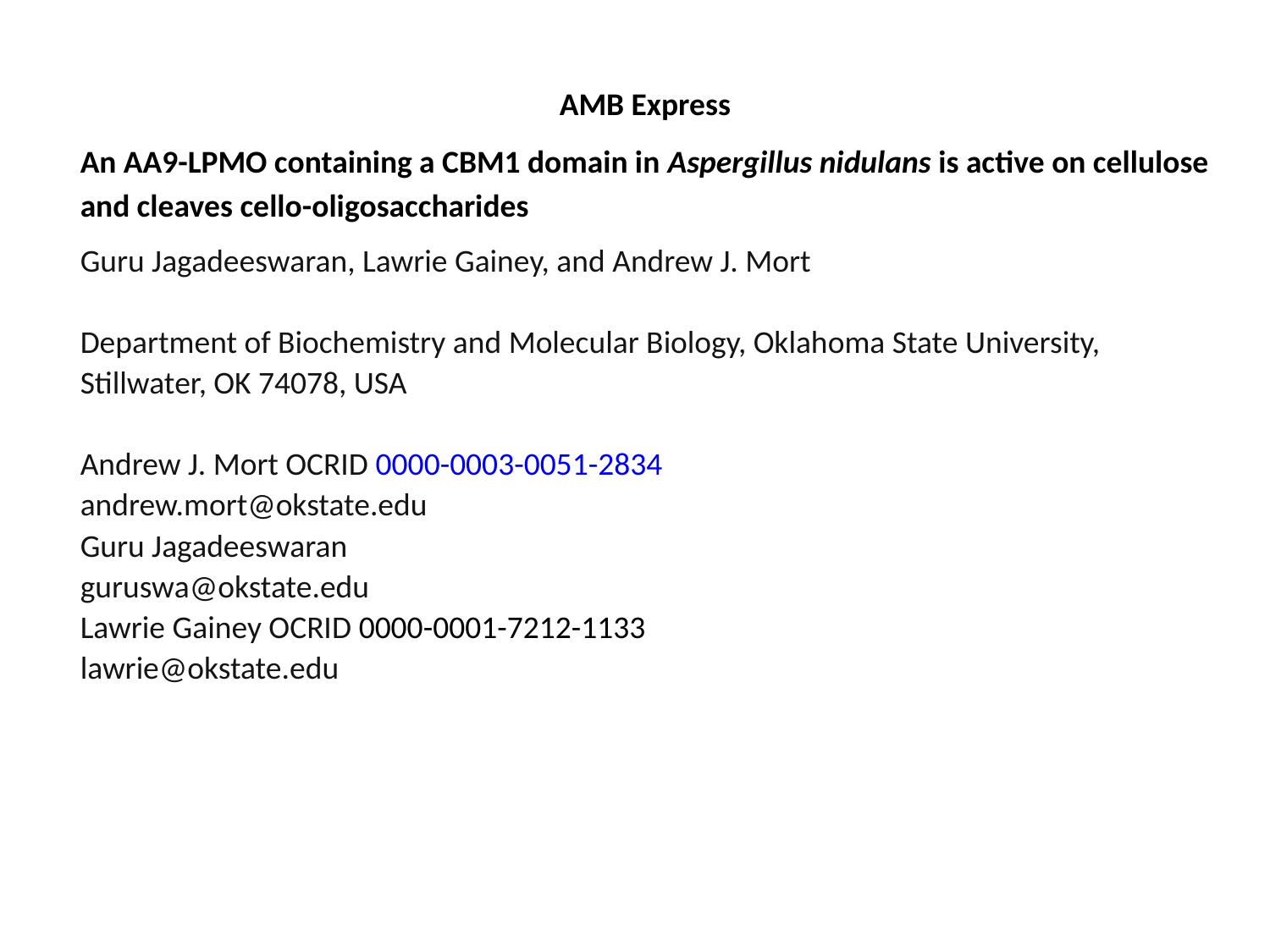

AMB Express
An AA9-LPMO containing a CBM1 domain in Aspergillus nidulans is active on cellulose and cleaves cello-oligosaccharides
Guru Jagadeeswaran, Lawrie Gainey, and Andrew J. Mort
Department of Biochemistry and Molecular Biology, Oklahoma State University, Stillwater, OK 74078, USA
Andrew J. Mort OCRID 0000-0003-0051-2834
andrew.mort@okstate.edu
Guru Jagadeeswaran
guruswa@okstate.edu
Lawrie Gainey OCRID 0000-0001-7212-1133
lawrie@okstate.edu

## Slide 2
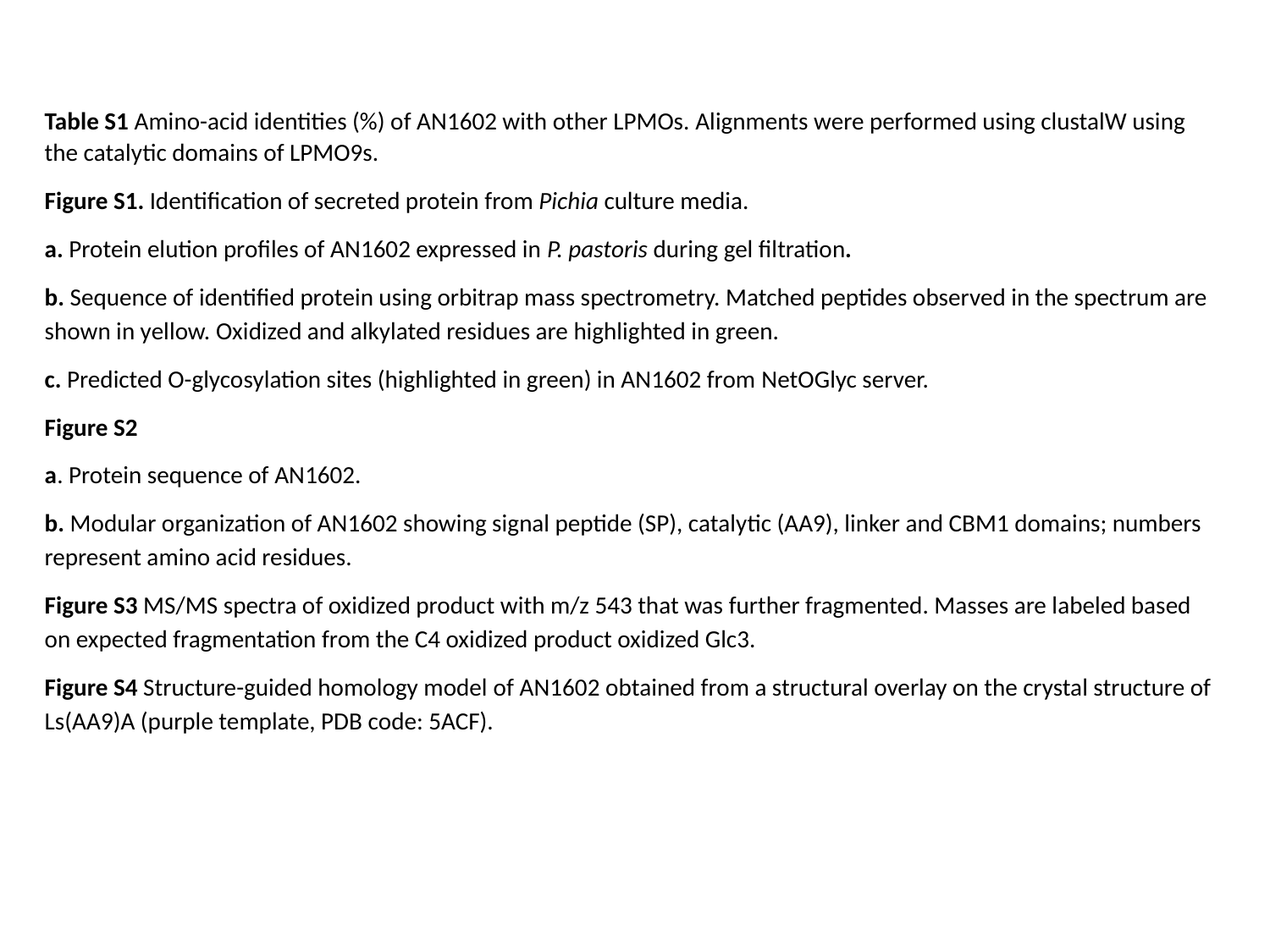

Table S1 Amino-acid identities (%) of AN1602 with other LPMOs. Alignments were performed using clustalW using the catalytic domains of LPMO9s.
Figure S1. Identification of secreted protein from Pichia culture media.
a. Protein elution profiles of AN1602 expressed in P. pastoris during gel filtration.
b. Sequence of identified protein using orbitrap mass spectrometry. Matched peptides observed in the spectrum are shown in yellow. Oxidized and alkylated residues are highlighted in green.
c. Predicted O-glycosylation sites (highlighted in green) in AN1602 from NetOGlyc server.
Figure S2
a. Protein sequence of AN1602.
b. Modular organization of AN1602 showing signal peptide (SP), catalytic (AA9), linker and CBM1 domains; numbers represent amino acid residues.
Figure S3 MS/MS spectra of oxidized product with m/z 543 that was further fragmented. Masses are labeled based on expected fragmentation from the C4 oxidized product oxidized Glc3.
Figure S4 Structure-guided homology model of AN1602 obtained from a structural overlay on the crystal structure of Ls(AA9)A (purple template, PDB code: 5ACF).

## Slide 3
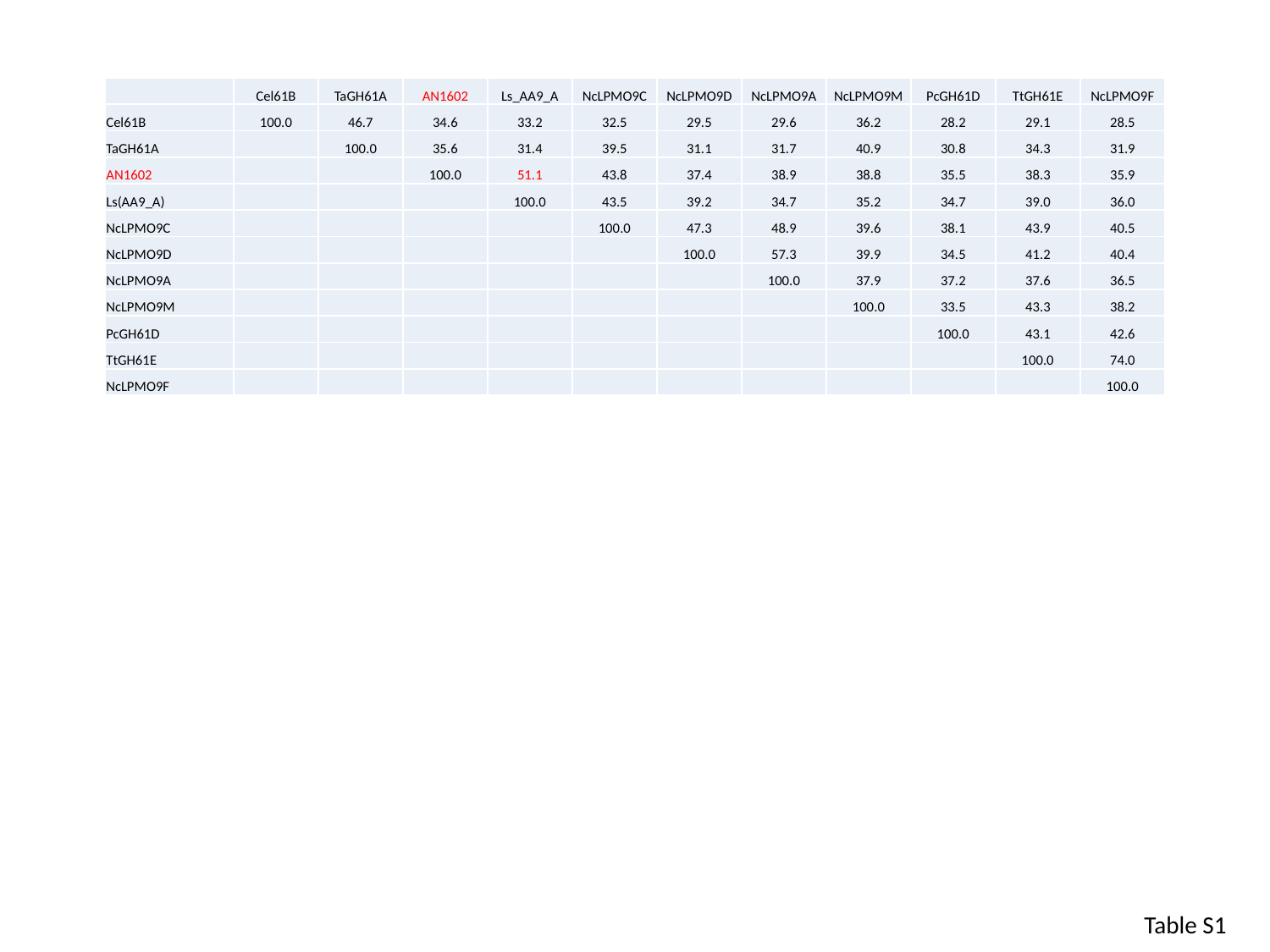

| | Cel61B | TaGH61A | AN1602 | Ls\_AA9\_A | NcLPMO9C | NcLPMO9D | NcLPMO9A | NcLPMO9M | PcGH61D | TtGH61E | NcLPMO9F |
| --- | --- | --- | --- | --- | --- | --- | --- | --- | --- | --- | --- |
| Cel61B | 100.0 | 46.7 | 34.6 | 33.2 | 32.5 | 29.5 | 29.6 | 36.2 | 28.2 | 29.1 | 28.5 |
| TaGH61A | | 100.0 | 35.6 | 31.4 | 39.5 | 31.1 | 31.7 | 40.9 | 30.8 | 34.3 | 31.9 |
| AN1602 | | | 100.0 | 51.1 | 43.8 | 37.4 | 38.9 | 38.8 | 35.5 | 38.3 | 35.9 |
| Ls(AA9\_A) | | | | 100.0 | 43.5 | 39.2 | 34.7 | 35.2 | 34.7 | 39.0 | 36.0 |
| NcLPMO9C | | | | | 100.0 | 47.3 | 48.9 | 39.6 | 38.1 | 43.9 | 40.5 |
| NcLPMO9D | | | | | | 100.0 | 57.3 | 39.9 | 34.5 | 41.2 | 40.4 |
| NcLPMO9A | | | | | | | 100.0 | 37.9 | 37.2 | 37.6 | 36.5 |
| NcLPMO9M | | | | | | | | 100.0 | 33.5 | 43.3 | 38.2 |
| PcGH61D | | | | | | | | | 100.0 | 43.1 | 42.6 |
| TtGH61E | | | | | | | | | | 100.0 | 74.0 |
| NcLPMO9F | | | | | | | | | | | 100.0 |
Table S1

## Slide 4
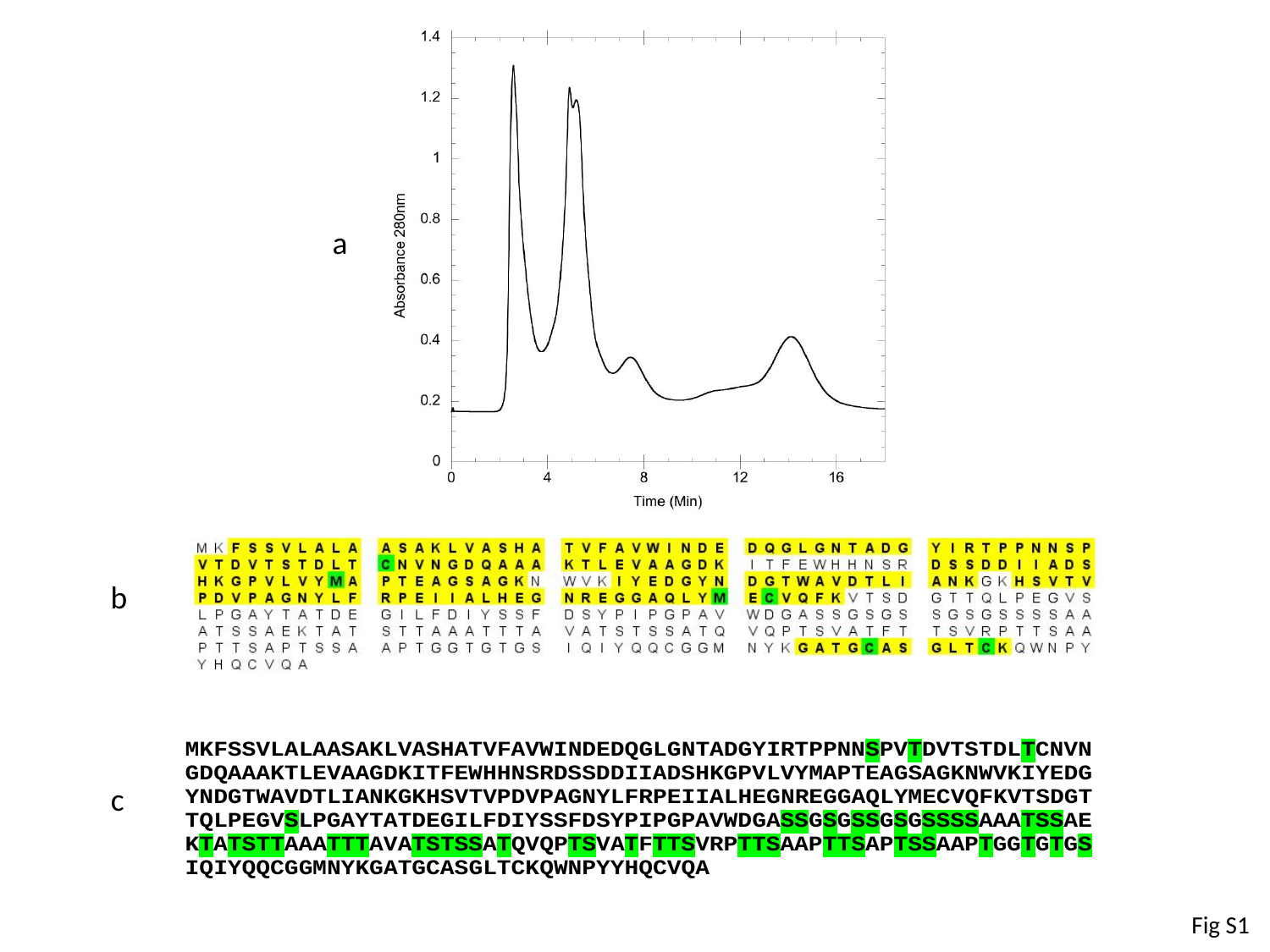

a
b
c
Fig S1

## Slide 5
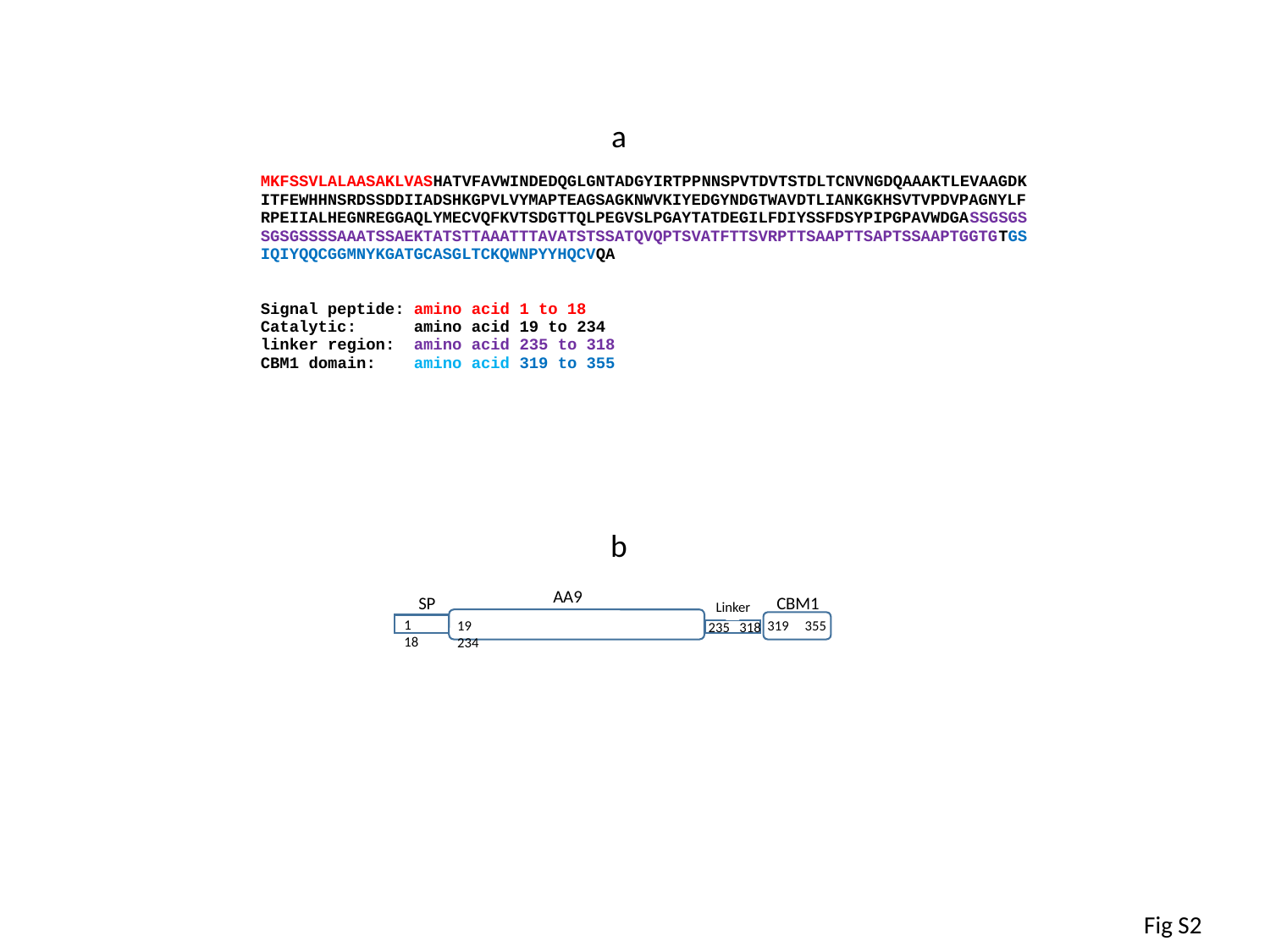

a
b
AA9
SP
CBM1
Linker
1 18
319 355
19 234
235 318
23
Fig S2

## Slide 6
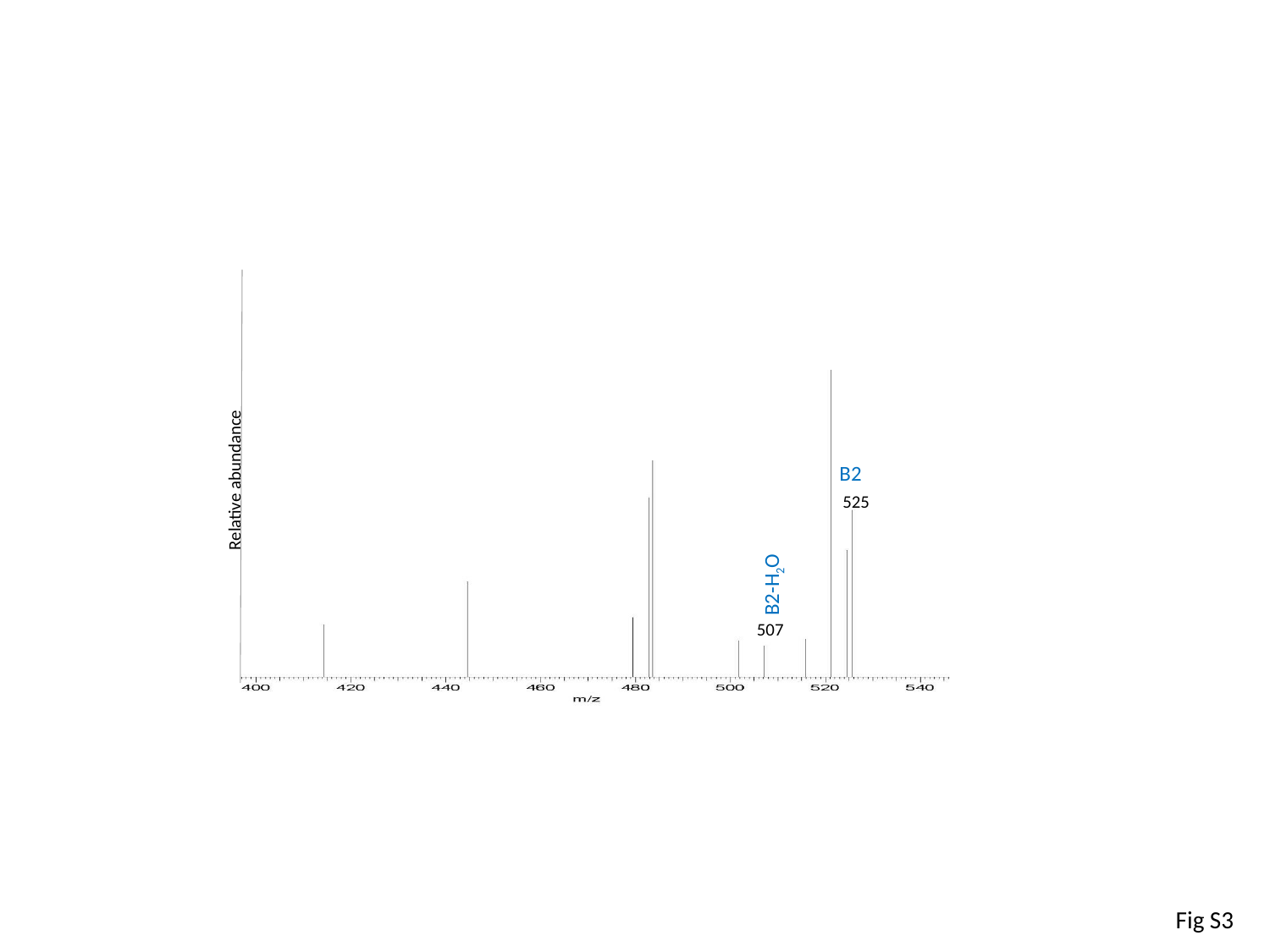

B2
Relative abundance
525
B2-H2O
507
Fig S3

## Slide 7
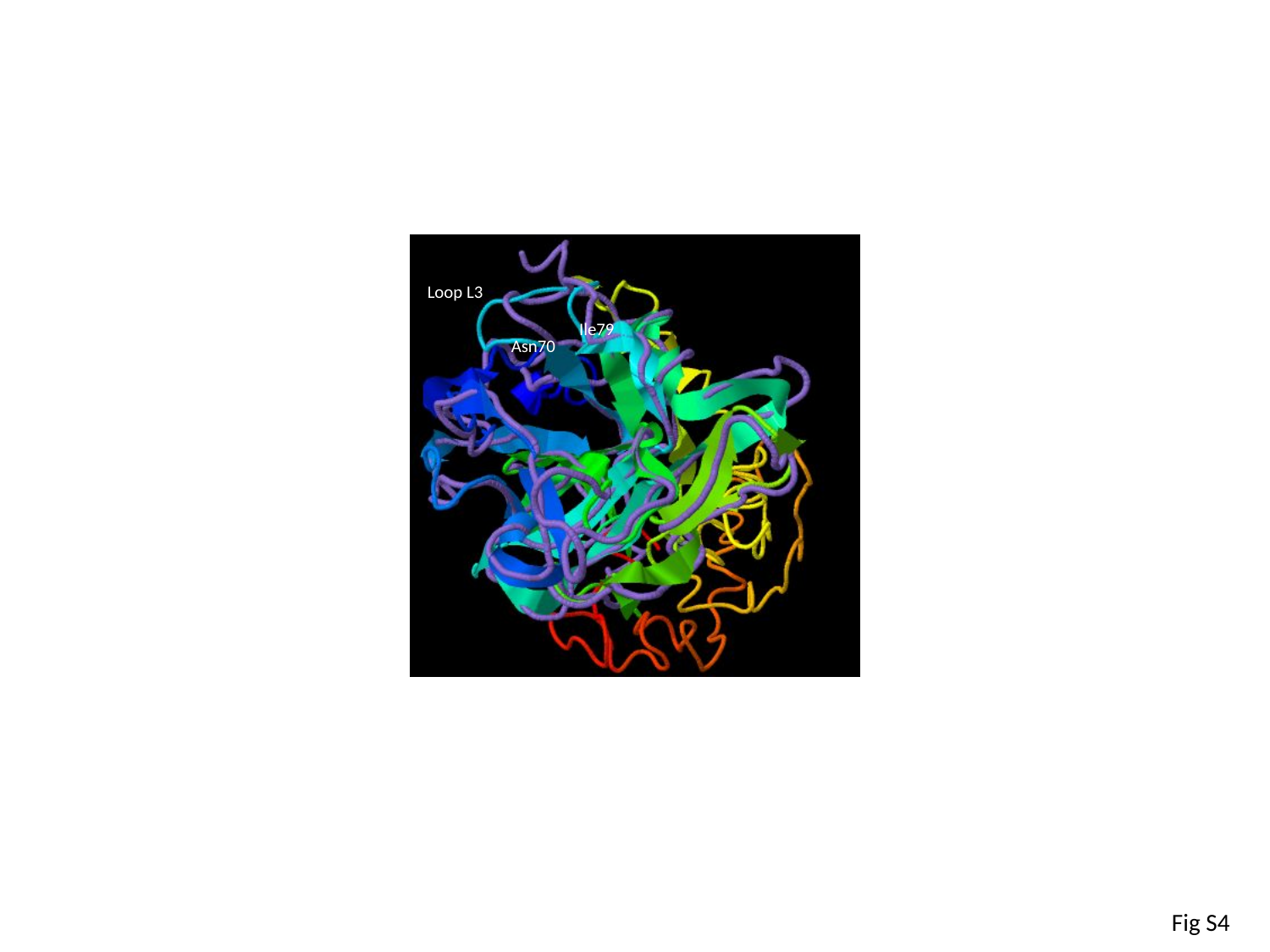

Loop L3
Ile79
Asn70
Fig S4
